# Association Between Years of Education and Amyloid Burden in Patients With Subjective Cognitive Decline, MCI, and Alzheimer Disease

**DOI:** 10.1212/WNL.0000000000208053

**Published:** 2024-02-20

**Authors:** Merle Hönig, Daniele Altomare, Camilla Caprioglio, Lyduine Collij, Frederik Barkhof, Bart Van Berckel, Philip Scheltens, Gill Farrar, Mark R. Battle, Hendrik Theis, Kathrin Giehl, Gerard N. Bischof, Valentina Garibotto, José Luis L. Molinuevo, Oriol Grau-Rivera, Julien Delrieu, Pierre Payoux, Jean Francois Demonet, Agneta K. Nordberg, Irina Savitcheva, Zuzana Walker, Paul Edison, Andrew W. Stephens, Rossella Gismondi, Frank Jessen, Christopher J. Buckley, Juan Domingo Gispert, Giovanni B. Frisoni, Alexander Drzezga, for the AMYPAD Consortium

**Affiliations:** From the Department of Nuclear Medicine, Faculty of Medicine and University Hospital Cologne (M.H., H.T., K.G., G.N.B., A.D.), University of Cologne; Institute of Neuroscience and Medicine (INM-2) (M.H., K.G., A.D.), Molecular Organization of the Brain, Forschungszentrum Jülich, Germany; Neurology Unit (D.A.), Department of Clinical and Experimental Sciences, University of Brescia, Italy; Laboratory of Neuroimaging of Aging (LANVIE) (D.A.), University of Geneva; Geneva Memory Center (D.A., C.C., G.B.F.), Geneva University Hospitals, Switzerland; Amsterdam UMC (L.C., F.B., B.V.B., P.S.), Location VUmc, Radiology; Amsterdam Neuroscience (L.C., F.B., B.V.B., P.S.), Brain Imaging, the Netherlands; Queen Square Institute of Neurology and Centre for Medical Image Computing (F.B.), University College London; GE Healthcare (G.F., M.R.B., C.J.B.), Pharmaceutical Diagnostics, Amersham, United Kingdom; Department of Neurology (H.T.), Faculty of Medicine and University Hospital Cologne, University of Cologne, Germany; Division of Nuclear Medicine and Molecular Imaging (V.G.), Diagnostic Department, University Hospitals of Geneva; Laboratory of Neuroimaging and Innovative Molecular Tracers (NIMTLab) (V.G.), Faculty of Medicine, Department of Radiology, University of Geneva; Center for Biomedical Imaging (CIBM) (V.G.), Geneva, Switzerland; Barcelonaβeta Brain Research Center (BBRC) (J.L.L.M., O.G.-R., J.D.G.), Pasqual Maragall Foundation, Barcelona, Spain; Gérontopôle (J.D., P.P., J.F.D.), Department of Geriatrics, Toulouse University Hospital; Maintain Aging Research Team (J.D.), CERPOP, Inserm, Université Paul Sabatier, Toulouse; ToNIC (P.P.), Toulouse NeuroImaging Center, Université de Toulouse, Inserm, UPS, France; Center for Alzheimer Research (A.K.N.), Department of Neurobiology, Care Sciences and Society, Karolinska Institutet; Theme Inflammation and Aging (A.K.N.), Karolinska University Hospital, Stockholm; Medical Radiation Physics and Nuclear Medicine (I.S.), Karolinska University Hospital, Sweden; Division of Psychiatry (Z.W.), University College London, London and Essex Partnership University NHS Foundation Trust; Department of Brain Sciences (P.E.), Imperial College London, United Kingdom; Life Molecular Imaging (A.W.S., R.G.), Berlin; Department of Psychiatry (F.J.), Faculty of Medicine and University Hospital Cologne, University of Cologne; and German Center for Neurodegenerative Diseases (DZNE) (F.J., A.D.), Bonn-Cologne, Germany.

## Abstract

**Objectives:**

Higher-educated patients with Alzheimer disease (AD) can harbor greater neuropathologic burden than those with less education despite similar symptom severity. In this study, we assessed whether this observation is also present in potential preclinical AD stages, namely in individuals with subjective cognitive decline and clinical features increasing AD likelihood (SCD+).

**Methods:**

Amyloid-PET information ([^18^F]Flutemetamol or [^18^F]Florbetaben) of individuals with SCD+, mild cognitive impairment (MCI), and AD were retrieved from the AMYPAD-DPMS cohort, a multicenter randomized controlled study. Group classification was based on the recommendations by the SCD-I and NIA-AA working groups. Amyloid PET images were acquired within 8 months after initial screening and processed with AMYPYPE. Amyloid load was based on global Centiloid (CL) values. Educational level was indexed by formal schooling and subsequent higher education in years. Using linear regression analysis, the main effect of education on CL values was tested across the entire cohort, followed by the assessment of an education-by-diagnostic-group interaction (covariates: age, sex, and recruiting memory clinic). To account for influences of non-AD pathology and comorbidities concerning the tested amyloid-education association, we compared white matter hyperintensity (WMH) severity, cardiovascular events, depression, and anxiety history between lower-educated and higher-educated groups within each diagnostic category using the Fisher exact test or χ^2^ test. Education groups were defined using a median split on education (Md = 13 years) in a subsample of the initial cohort, for whom this information was available.

**Results:**

Across the cohort of 212 individuals with SCD+ (M(Age) = 69.17 years, *F* 42.45%), 258 individuals with MCI (M(Age) = 72.93, *F* 43.80%), and 195 individuals with dementia (M(Age) = 74.07, *F* 48.72%), no main effect of education (ß = 0.52, 95% CI −0.30 to 1.58), but a significant education-by-group interaction on CL values, was found (*p* = 0.024) using linear regression modeling. This interaction was driven by a negative association of education and CL values in the SCD+ group (ß = −0.11, 95% CI −4.85 to −0.21) and a positive association in the MCI group (ß = 0.15, 95% CI 0.79–5.22). No education-dependent differences in terms of WMH severity and comorbidities were found in the subsample (100 cases with SCD+, 97 cases with MCI, 72 cases with dementia).

**Discussion:**

Education may represent a factor oppositely modulating subjective awareness in preclinical stages and objective severity of ongoing neuropathologic processes in clinical stages.

## Introduction

In Alzheimer disease (AD), longer formal education (i.e., higher education) is not only related to lower disease prevalence but also to greater tolerance against the effects of neuropathology on functionality. Given these observations, the concepts of resistance (i.e., withstanding neuropathology buildup) and resilience (i.e., coping with neuropathology) have been established.^1^ Studies investigating resilience mechanisms reported that better-educated patients with mild cognitive impairment (MCI) and dementia harbor greater amyloid^2^ and tau^3^ burden at the diagnosis compared with lower-educated counterparts with similar clinical impairment. Thus, higher education seems to diminish symptom severity and/or delay the onset of objectively symptomatic disease. The protective effects of education on AD pathology in potential preclinical stages of AD, namely subjective cognitive decline (SCD), have recently become of interest.

SCD occurs in approximately 25% of individuals older than 60 years but cannot be detected by standard neuropsychological tests.^4^ This concept can be extended to SCD plus (SCD+), which refers to additional features increasing AD likelihood.^4^ Of interest, recent studies demonstrated that higher education was associated with slower cognitive decline in individuals with SCD.^5^ It remains, however, unknown whether higher-educated individuals with SCD present greater neuropathologic burden compared with those with lower education. In this study, we examined the relationship between education and amyloid plaques, the characteristic AD neuropathologic hallmark, using PET imaging in individuals with SCD+, MCI, and dementia.

## Methods

### Participants

We used data of the AMYPAD-DPMS cohort, a phase 4, multicenter, prospective randomized controlled study,^6^ which aimed testing the clinical utility of amyloid PET. Participants presented cognitive complaints and were recruited from 8 European memory clinics. Subjects were randomized into 3 arms: early amyloid-PET (within 1 month from baseline), late amyloid-PET (8 months after baseline), or free choice (if and when it was requested by the managing physician). Classification into SCD+, MCI, or dementia was based on current recommendations by the SCD-I working group^4^ and NIA-AA^7,8^. The most relevant features defining SCD+ were age 60–85 years, perceived memory decline within previous 5 years and duration >6 months, Mini-Mental State Examination (MMSE) > 24, exclusion of MCI, explicit concerns, and active seeking of consultation. According to the AMYPAD-DPMS inclusion criteria, age limit of patients with MCI and dementia was between 50 and 85 years; however, in this study, we included only those older than 60 years to match the age inclusion criterion of the SCD+ group (Table 1).

**Table 1:** Inclusion Criteria and Sample Size of the Selected Cohort

	Initial Sample (N = 840)	→	Evaluable PET scan	→	Available MMSE and >24 for SCD+	→	Age range 60–85 y
SCD+	n = 244		n = 219		n = 215		n = 212
MCI	n = 341		n = 290		n = 287		n = 258
Dementia	n = 255		n = 215		n = 213		n = 195

Abbreviation: MMSE = Mini-Mental State Examination.

### Standard Protocol Approvals, Registrations, and Patient Consents

The ethics committees of all recruiting clinics approved the study. All participants gave written informed consent. The trial was registered at EudraCT (2017-002527-21).

### Biographical and Cognitive Data

Educational level was defined as formal schooling plus subsequent higher education in years. Cognitive performance (i.e., MMSE) and medical history (i.e., history of depression, anxiety or cardiovascular events, and hypertension) were acquired at the baseline visit.

#### Amyloid PET Imaging

For all patients, either a [^18^F]Flutemetamol or [^18^F]Florbetaben scan was available. PET acquisition consisted of 4 frames (4 × 5 minutes), acquired 90–110 minutes post injection. Because MRI was not routinely available, PET images were processed and quantified with AMYPYPE, which is a modified version of GE Healthcare's CortexID software. After spatial normalization to MNI space and intensity standardization using the whole cerebellum, the normalized PET scans were converted to the Centiloid (CL) scale allowing pooled analysis across tracer types.^7^ Global CL values were used for subsequent analyses.

#### White Matter Hyperintensities (WMH)

For a subset, structural MRI scans were obtained shortly after the baseline visit and rated by trained experts regarding the WMH severity using the Fazekas score.^9^

### Statistical Analysis

To test the association between educational attainment and amyloid load, linear regression was used to first test the main effect of education on CL values correcting for age, sex, diagnostic group, and recruiting memory clinic. Next, an education-by-diagnostic-group interaction term was introduced to the regression model using dummy coding on diagnostic group. Underlying assumptions for regression were investigated (e.g., homoscedasticity and residual normality).

To account for potential influences of non-AD pathology and comorbidities concerning the education-amyloid association, we assessed education-dependent differences of WMH severity, hypertension, and history of depression or anxiety, cardiovascular event in a subgroup of the studied cohort. Given the variables' ordinal nature, we split the cohort into a higher-educated (>Md) and lower-educated (≤Md) group based on a median split on education (Md = 13 years). The Fisher exact test was used for the Fazekas score (due to observations n < 5) and χ^2^ tests for the remaining comorbidity variable comparisons between education groups within and across diagnostic groups.

All analyses were performed using SPSS version 28. R Studio version 4.1.2 (package: ggplot2_3.3.6) was used for illustration.

### Data Availability

Data not provided in the article because of space limitations may be shared (anonymized) at the request of any qualified investigator.

## Results

Results are based on a total of 212 individuals with SCD+ (M(Age) = 69.17 ± 5.96, *F* 42.45%), 258 individuals with MCI (M(Age) = 72.93 ± 6.19, *F* 43.80%), and 195 individuals with dementia (M(Age) = 74.07 ± 6.33, *F* 48.72%) (Table 2). The SCD+ group was significantly younger (H(2) = 65.27, Eta^2^ = 0.098), had higher MMSE scores (H(2) = 325.89, Eta^2^ = 0.491) and education (H(2) = 35.11, Eta^2^ = 0.053), and lower CL values (H(2) = 73.94, Eta^2^ = 0.111) than the MCI and dementia groups. Proportion of sex and tracer type was similar across groups. For detailed information on the subgroup (100 cases with SCD+, 97 with MCI, and 72 with dementia), please see the supplements (eAppendix 1, links.lww.com/WNL/D364).

**Table 2:** Demographic Characteristics of the Cohort Are Reported (Mean and SD)

	SCD+, n = 212	MCI, n = 258	Dementia, n = 195
Age	69.17 (5.96)	72.93 (6.19)	74.07 (6.33)
Sex (M/F)	122/90	145/113	100/95
MMSE	28.74 (1.31)	25.89 (3.02)	21.82 (4.37)
Education	14.02 (3.46)	12.59 (3.94)	11.75 (3.95)
Tracer FBB/FMT	87/125	129/129	101/94
Global Centiloid	23.33 (37.97)	46.58 (46.83)	66.25 (49.68)

Abbreviations: FBB = Florbetaben; FMT = Flutemetamol; MMSE = Mini-Mental State Examination.

The results of the linear regression yielded no main effect of education (ß = 0.52, 95% CI −0.30–1.58) but a significant education-by-diagnostic-group interaction on CL values (*p* = 0.024). With increasing education, the SCD+ group presented lower CL values (ß = −0.11, 95% CI −4.85 to −0.21) relative to the dementia group. By contrast, in the MCI group, higher education was linked to an increase in CL values (ß = 0.15, 95% CI 0.79–5.22) relative to the SCD+ group (Figure). The effect sizes were overall rather small. In the subgroup, no education-dependent differences were observed for WMH severity and other comorbidities per diagnostic group (see eAppendix 1, links.lww.com/WNL/D364 for statistical summary).

**Figure:**
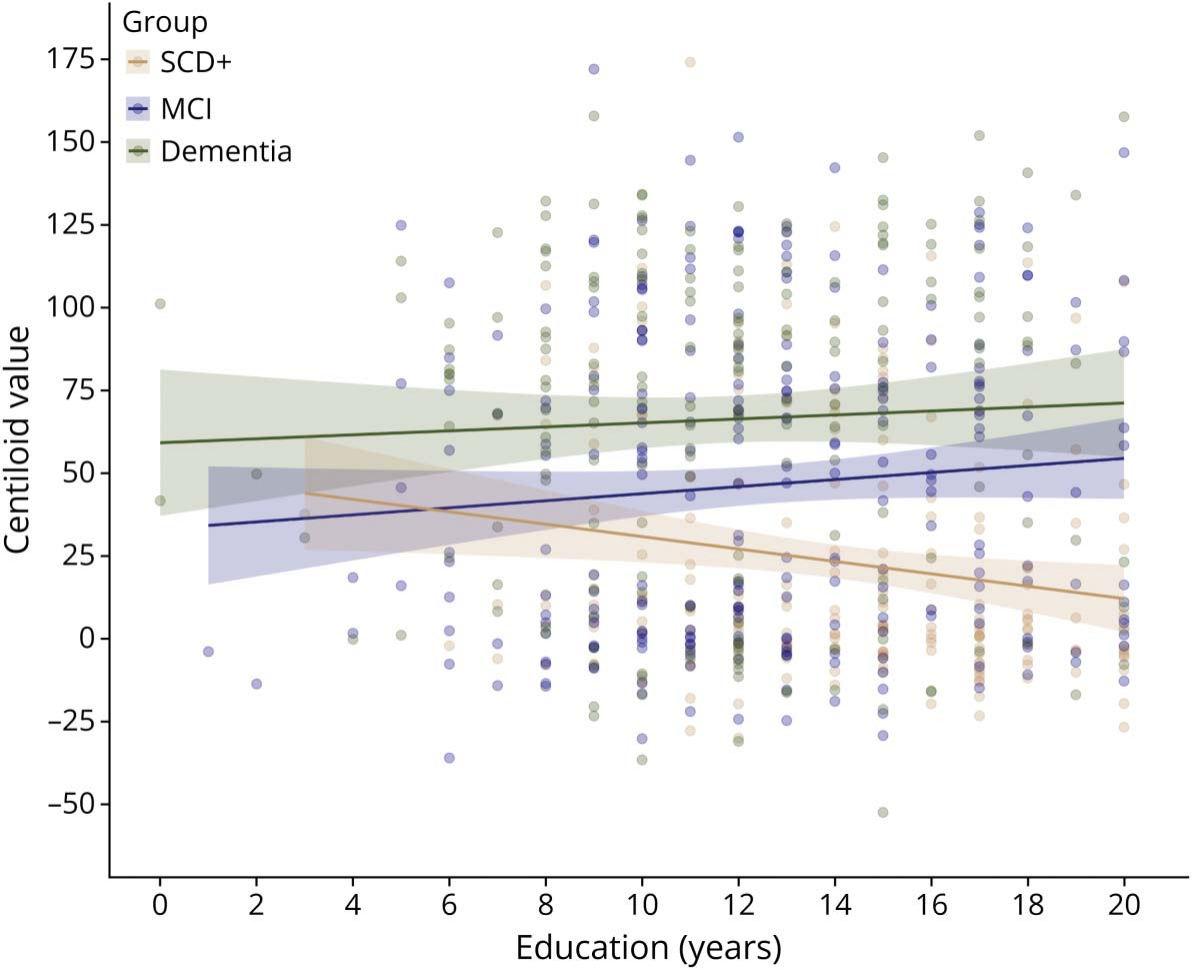
Association Between Years of Education and Centiloid Value for Potential Preclinical Stages (SCD+) and Clinical Stages (MCI and Dementia) of AD

## Discussion

The findings indicate that higher-educated individuals in potential preclinical stages of AD (i.e., SCD+) may be more perceptive of the effects of subthreshold AD pathology on subjective functioning. By contrast, in clinical AD stages, higher educational attainment seems to be associated with a greater tolerance to the effects of marked amyloid burden on objective cognitive measures. The latter observation is consistent with previous findings,^2^ arguing that higher-educated patients with AD obtain resilience mechanisms, such as compensatory network recruitment strategies, supporting the prolongation of functional decline despite increasing neuropathologic burden.

By contrast, the observed inverse association in the SCD+ group could overall be explained by greater alertness and concern regarding their cognitive well-being and better access to health care systems. These factors may concomitantly contribute to the early diagnosis of SCD+, even at subthreshold levels of AD pathology. Aside from this, higher education may be linked to resistance mechanisms, slowing pathology accumulation and thus disease progression. Indeed, the onset of MCI in cases with SCD was reported to be delayed by 9 years in individuals with higher premorbid intelligence.^10^ Yet, due to the cross-sectional nature of this study, this assumption of resistance mechanisms cannot be readily answered, and longitudinal designs are warranted. Moreover, we cannot rule out that better educated individuals with SCD+ may have additionally experienced from other pathologies than AD. Yet, in terms of WMH and other comorbidities, we did not observe an education effect.

Overall, the results indicate that education may have opposing modulatory effects depending on the disease stage. In potential preclinical stages, higher education may raise subjective awareness for ongoing neurodegenerative disease and may facilitate resistance mechanisms, while in clinical stages, higher education supports attenuation of objective symptomatic severity likely through resilience mechanisms.

## Appendix 1 Authors

**Table:**

Name	Location	Contribution
Merle Hönig, PhD	Department of Nuclear Medicine, Faculty of Medicine and University Hospital Cologne, University of Cologne; Institute of Neuroscience and Medicine (INM-2), Molecular Organization of the Brain, Forschungszentrum Jülich, Germany	Drafting/revision of the article for content, including medical writing for content; study concept or design; and analysis or interpretation of data
Daniele Altomare, PhD	Neurology Unit, Department of Clinical and Experimental Sciences, University of Brescia, Italy; Laboratory of Neuroimaging of Aging (LANVIE), University of Geneva; Geneva Memory Center, Geneva University Hospitals, Switzerland	Drafting/revision of the article for content, including medical writing for content; major role in the acquisition of data; study concept or design; and analysis or interpretation of data
Camilla Caprioglio, MSc	Geneva Memory Center, Geneva University Hospitals, Switzerland	Major role in the acquisition of data; study concept or design
Lyduine Collij, PhD	Amsterdam UMC, Location VUmc, Radiology; Amsterdam Neuroscience, Brain Imaging, the Netherlands	Drafting/revision of the article for content, including medical writing for content; major role in the acquisition of data
Frederik Barkhof, MD	Amsterdam UMC, Location VUmc, Radiology; Amsterdam Neuroscience, Brain Imaging, the Netherlands; Queen Square Institute of Neurology and Centre for Medical Image Computing, University College London, United Kingdom	Drafting/revision of the article for content, including medical writing for content; major role in the acquisition of data; study concept or design; and analysis or interpretation of data
Bart Van Berckel, MD	Amsterdam UMC, Location VUmc, Radiology; Amsterdam Neuroscience, Brain Imaging, the Netherlands	Major role in the acquisition of data; study concept or design
Philip Scheltens, MD, PhD	Amsterdam UMC, Location VUmc, Radiology; Amsterdam Neuroscience, Brain Imaging, the Netherlands	Major role in the acquisition of data; study concept or design
Gill Farrar, PhD	GE Healthcare, Pharmaceutical Diagnostics, Amersham, United Kingdom	Major role in the acquisition of data; study concept or design
Mark R. Battle, BSc	GE Healthcare, Pharmaceutical Diagnostics, Amersham, United Kingdom	Major role in the acquisition of data; study concept or design
Hendrik Theis, MD	Department of Nuclear Medicine; Department of Neurology, Faculty of Medicine and University Hospital Cologne, University of Cologne, Germany	Drafting/revision of the article for content, including medical writing for content; analysis or interpretation of data
Kathrin Giehl, PhD	Department of Nuclear Medicine, Faculty of Medicine and University Hospital Cologne, University of Cologne; Institute of Neuroscience and Medicine (INM-2), Molecular Organization of the Brain, Forschungszentrum Jülich, Germany	Major role in the acquisition of data; study concept or design
Gerard N. Bischof, PhD	Department of Nuclear Medicine, Faculty of Medicine and University Hospital Cologne, University of Cologne, Germany	Study concept or design; analysis or interpretation of data
Valentina Garibotto, MD	Division of Nuclear Medicine and Molecular Imaging, Diagnostic Department, University Hospitals of Geneva; Laboratory of Neuroimaging and Innovative Molecular Tracers (NIMTLab), Faculty of Medicine, Department of Radiology, University of Geneva; Center for Biomedical Imaging (CIBM), Geneva, Switzerland	Drafting/revision of the article for content, including medical writing for content; major role in the acquisition of data; and study concept or design
José Luis L. Molinuevo, MD, PhD	Barcelonaβeta Brain Research Center (BBRC), Pasqual Maragall Foundation, Barcelona, Spain	Drafting/revision of the article for content, including medical writing for content; major role in the acquisition of data; and study concept or design
Oriol Grau-Rivera, MD	Barcelonaβeta Brain Research Center (BBRC), Pasqual Maragall Foundation, Barcelona, Spain	Drafting/revision of the article for content, including medical writing for content; major role in the acquisition of data; and study concept or design
Julien Delrieu	Gérontopôle, Department of Geriatrics, Toulouse University Hospital; Maintain Aging Research team, CERPOP, Inserm, Université Paul Sabatier, Toulouse, France	Drafting/revision of the article for content, including medical writing for content; major role in the acquisition of data
Pierre Payoux, MD, PhD	Gérontopôle, Department of Geriatrics, Toulouse University Hospital; ToNIC, Toulouse NeuroImaging Center, Université de Toulouse, Inserm, UPS, France	Drafting/revision of the article for content, including medical writing for content; major role in the acquisition of data; and study concept or design
Jean Francois Demonet, MD, PhD	Gérontopôle, Department of Geriatrics, Toulouse University Hospital, France	Major role in the acquisition of data; study concept or design
Agneta Karin Nordberg, MD, PhD	Center for Alzheimer Research, Department of Neurobiology, Care Sciences and Society, Karolinska institutet; Theme Inflammation and Aging, Karolinska University Hospital, Stockholm, Sweden	Drafting/revision of the article for content, including medical writing for content; major role in the acquisition of data; and study concept or design
Irina Savitcheva, MD, PhD	Medical Radiation Physics and Nuclear Medicine, Karolinska University Hospital, Sweden	Major role in the acquisition of data; study concept or design
Zuzana Walker, MD	Division of Psychiatry, University College London, London and Essex Partnership University NHS Foundation Trust, United Kingdom	Drafting/revision of the article for content, including medical writing for content; major role in the acquisition of data; and study concept or design
Paul Edison, MD, PhD	Department of Brain Sciences, Imperial College London, United Kingdom	Drafting/revision of the article for content, including medical writing for content; major role in the acquisition of data; and study concept or design
Andrew W. Stephens, MD, PhD	Life Molecular Imaging, Berlin, Germany	Major role in the acquisition of data; study concept or design
Rossella Gismondi, MD	Life Molecular Imaging, Berlin, Germany	Drafting/revision of the article for content, including medical writing for content; major role in the acquisition of data; and study concept or design
Frank Jessen, MD	Department of Psychiatry, Faculty of Medicine and University Hospital Cologne, University of Cologne; German Center for Neurodegenerative Diseases (DZNE), Bonn-Cologne, Germany	Major role in the acquisition of data; study concept or design
Christopher J. Buckley, PhD	GE Healthcare, Pharmaceutical Diagnostics, Amersham, United Kingdom	Major role in the acquisition of data; study concept or design
Juan Domingo Gispert, PhD	Barcelonaβeta Brain Research Center (BBRC), Pasqual Maragall Foundation, Barcelona, Spain	Major role in the acquisition of data; study concept or design
Giovanni B. Frisoni, MD	Geneva Memory Center, Geneva University Hospitals, Switzerland	Drafting/revision of the article for content, including medical writing for content; major role in the acquisition of data; study concept or design; and analysis or interpretation of data
Alexander Drzezga, MD	Department of Nuclear Medicine, Faculty of Medicine and University Hospital Cologne, University of Cologne; Institute of Neuroscience and Medicine (INM-2), Molecular Organization of the Brain, Forschungszentrum Jülich; German Center for Neurodegenerative Diseases (DZNE), Bonn-Cologne, Germany	Drafting/revision of the article for content, including medical writing for content; major role in the acquisition of data; study concept or design; and analysis or interpretation of data

## Appendix 2 Coinvestigators

**Table:**

Coinvestigators are listed at links.lww.com/WNL/D365.
